# Downregulation of miR-16 via URGCP pathway contributes to glioma growth

**DOI:** 10.1038/s41598-017-14035-2

**Published:** 2017-10-18

**Authors:** Liang Hong, Ouyang Qing, Zhou Ji, Zhang Chengqu, Chen Ying, Cui Hao, Xu Minhui, Xu Lunshan

**Affiliations:** 1 Department of Neurosurgery, Daping Hospital & Research Institute of Surgery, Third Military Medical University, Chongqing, 400042 China; 2 Department of Neurosurgery, the General Hospital of Rocket Army, Beijing, 100088 China; 3 Department of General Surgery and Center of Minimal Invasive Gastrointestinal Surgery, Southwest Hospital, Third Military Medical University, Chongqing, 400038 China

## Abstract

Experimental and clinical evidence points to a critical role of Upregulator of cell proliferation (URGCP/URG4) in controlling the progression of multiple tumors. However, the oncogenic role of URGCP in glioma still remains elusive. In this study we tried to investigate the oncogenic roles and molecular mechanisms of URGCP in glioma. We found that the levels of URGCP were upregulated in glioma, and that the high-levels of URGCP indicated a worse prognosis in glioma patients. URGCP and miR-16 are critical for glioma growth: silencing URGCP (shURGCP) inhibited glioma growth, while, the shURGCP-mediated proliferative inhibition could be recovered by antagonizing miR-16 (anta-miR-16) *in vivo* and *in vitro*. Mechanically, URGCP repressed miR-16 expression via activating NF-κB/c-myc pathway in glioma; Cyclins D1 and Cyclin E1 were identified as the direct targets of miR-16, thus, URGCP-mediated miR-16 downregulation accelerated cell proliferation by upregulating Cyclin D1 and Cyclin E1 expression. All these results suggested that URGCP accelerates glioma growth through the NF-κB/c-myc/miR-16/Cyclin D1/E1 pathway, and both URGCP and miR-16 function as a novel cell cycle regulators in glioma and could be considered as potential targets for glioma therapy.

## Introduction

Glioma is one of the most malignant central nerve system cancers with poor prognosis, various therapeutic strategies for glioma have been developed, including surgery, radiotherapy and chemotherapy, but no obvious improvements have been obtained^1,2^. Cancer is a disease characterized by abnormal cell cycle phases, thus, intensive study of the regulatory mechanisms of cell proliferation and cell cycle might provide new diagnostic and therapeutic approaches to glioma.

Upregulator of cell proliferation (URGCP/URG4, GenBank accession no. NM_017920), a novel gene located on chromosome 7p13, is originally identified as one of eight genes upregulated by hepatitis B virus X antigen (HBxAg) transduction in hepatocellular carcinoma cell line (HepG2)^3^. Experimental and clinical evidence points to a strong expression of URGCP in multiple cancers, including hepatocellular carcinoma, gastric cancer, osteosarcoma, and epithelial ovarian cancer^4–8^. URGCP has been shown to play a critical role in controlling proliferation, invasiveness, apoptotic resistance, and angiogenesis of tumors^4,8–10^. Furthermore, multiple findings have confirmed that URGCP supports sustained *in vivo* and *in vitro* growth of hepatocellular carcinoma by inducing a decrease in p27^Kip1^ and p21^Cip1^ levels and an increase in Cyclin D1 expression^4,11^. Notably, URGCP has been indentified to act as a potential diagnostic biomarker and immunotherapeutic target for glioma patients^12^. However, the bio-functions and molecular mechanisms of URGCP in the progression of glioma warrant further investigation.

MicroRNAs (miRNAs) are a class of non-coding, endogenous RNAs with regulatory functions^13^. It has been shown that miRNAs are aberrantly expressed in glioma, and they are shown to be involved in different stages of glioma cell cycle regulation^14,15^. Hsa-miR-16 belongs to the miR-15/miR-16 cluster that is located on the non-coding gene deleted in Leukemia 2 (DLEU2)^16^. Validated targets of miR-16 including many genes related to the control of cell cycle progression, such as Cyclin D1 and Cyclin E1^17,18^. It has been shown that overexpression of URGCP causes a change in the expression of miRNAs in hepatocellular carcinoma cells, and many miRNAs are involved in hepatocellular carcinoma progression by their regulation of different signaling pathways^19^, suggesting that URGCP-mediated miRNAs are potent regulators of protein expression and cell fate that act on multiple levels in tumor growth. However, at present, no work has assessed the relationship between URGCP and miRNAs in glioma.

Despite the undeniable role of URGCP in tumor progression, the bio-functions of URGCP and URGCP-mediated miRNAs have not been explored in glioma. Here, our study indicates that URGCP promotes glioma growth through upregulating Cyclin D1 and Cyclin E1 expression, and the NF-κB/c-myc/miR-16 pathway is involved in URGCP-mediated Cyclin D1 and Cyclin E1 overexpression in glioma.

## Results

### URGCP is overexpressed in glioma tissues and glioma cell lines

**Figure 1:**
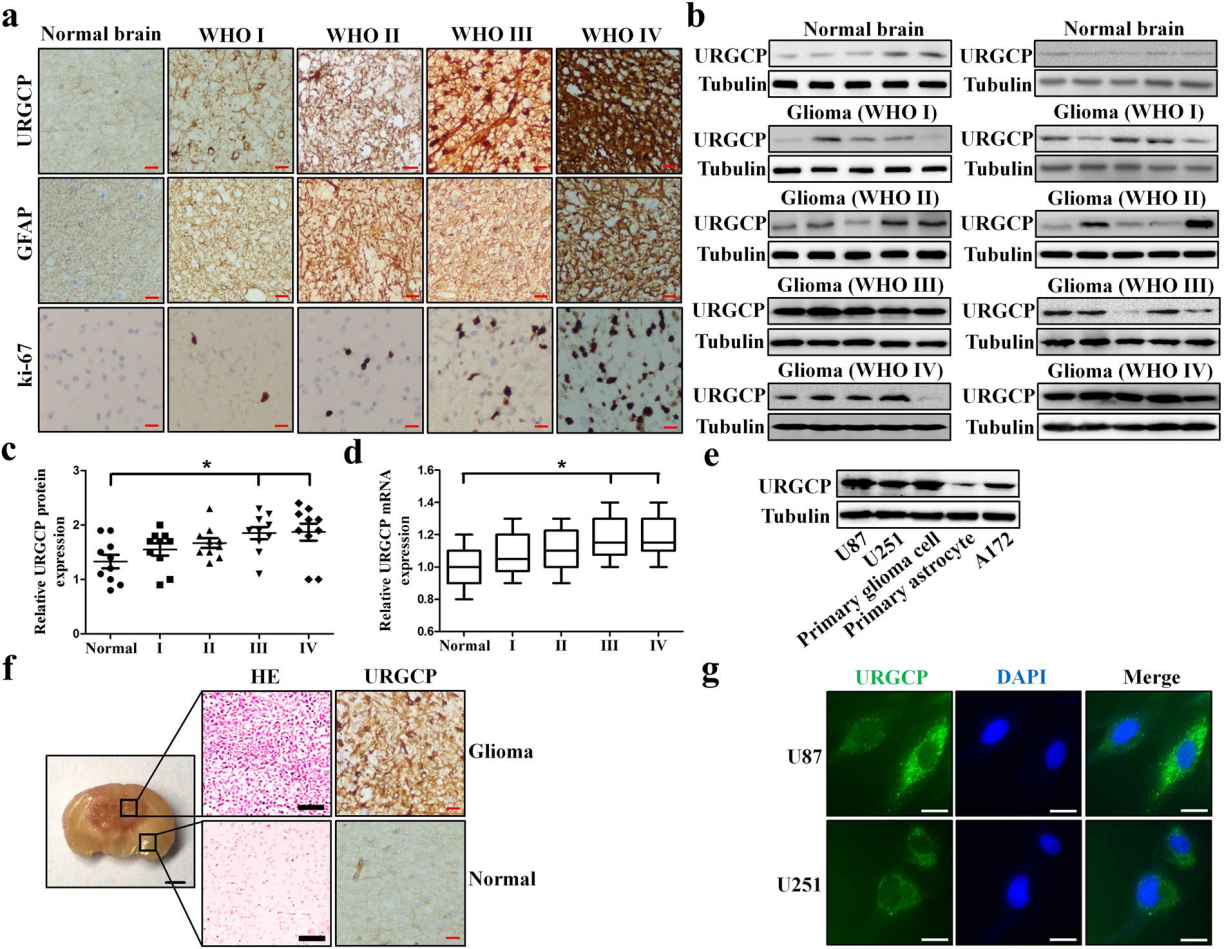
URGCP expression is elevated in glioma. (**a**) Representative images of URGCP, GFAP and Ki-67 staining using IHC assay in non-tumoral brain tissue and glioma specimens at different clinical stages. Scale bars: 50 μm. (**b**) Western blotting analysis of URGCP protein expression in 40 glioma specimens with different clinical stages and 10 non-tumoral brain tissues. (**c**) Scatter gram of URGCP expression quantification of western blotting in 40 glioma specimens with different histological grades, *p < 0.01. (**d**) Box plot of URGCP mRNA expression quantification of RT-qPCR in 40 glioma specimens with different histological grades, *p < 0.01. (**e**) Expression of URGCP protein in primary astrocytes, primary glioma cells and glioma cell lines (U251, U87 and A172). (**f**) URGCP expression in tumor core and relatively normal tissue of around U87-glioma were detected by IHC and HE counter stain. Scale bars: 1 mm (general) and 50 μm (HE and IHC). (**g**) The localization of URGCP in glioma cells were detected by immunofluorescence staining. Scale bar = 10 μm.

To detect the expression levels of URGCP in glioma specimens, Immunohistochemistry (IHC) was employed. As shown in Fig. 1a, URGCP immunostaining was only slightly detectable in non-tumoral brain tissue but were differentially upregulated in glioma tissues at distinct clinical stages. Western blotting and RT-qPCR assays also showed that both the protein and mRNA expression levels of URGCP were significantly upregulated in glioma tissues, compared to that of normal brain tissues (Fig. 1b–d). The Sun, Harrisand Hegidataset from R2 microarray platform, includes normal brain tissues and glioma samples with different histological grades, also indicated that the expression levels of URGCP were gradually increased in low-grade glioma tissues (II) and became markedly higher in high-grade glioma tissues (III and IV) (Supplementary Fig. 1a). Kaplan-Meier analysis of the overall survival for these dataset showed that high-levels of URGCP were associated with poor prognosis, whereas low-levels of URGCP indicated a good outcome (Supplementary Fig. 1b).

Next, we examined the expression levels of URGCP in glioma cell lines, western blotting revealed that the protein levels of URGCP were markedly higher in U87, U251, A172 and primary glioma cells, compared to primary astrocytes (Fig. 1e). We also determined URGCP levels *in vivo* by transplanting U87 cells into the brains of nude mice to establish an orthotopic graft model. As shown in Fig. 1f, the expression level of URGCP was markedly higher in orthotopic glioma compared to non-tumoral brain tissue. Finally, to visualize the subcell location of URGCP in glioma cells, immunofluorescence staining was performed. As shown in Fig. 1g, URGCP was consistently localized to the cytosol in U87 and U251 cells. Taken together, these data suggest that the expression levels of URGCP are widely upregulated in glioma.

### URGCP enhances glioma cell proliferation and growth

**Figure 2:**
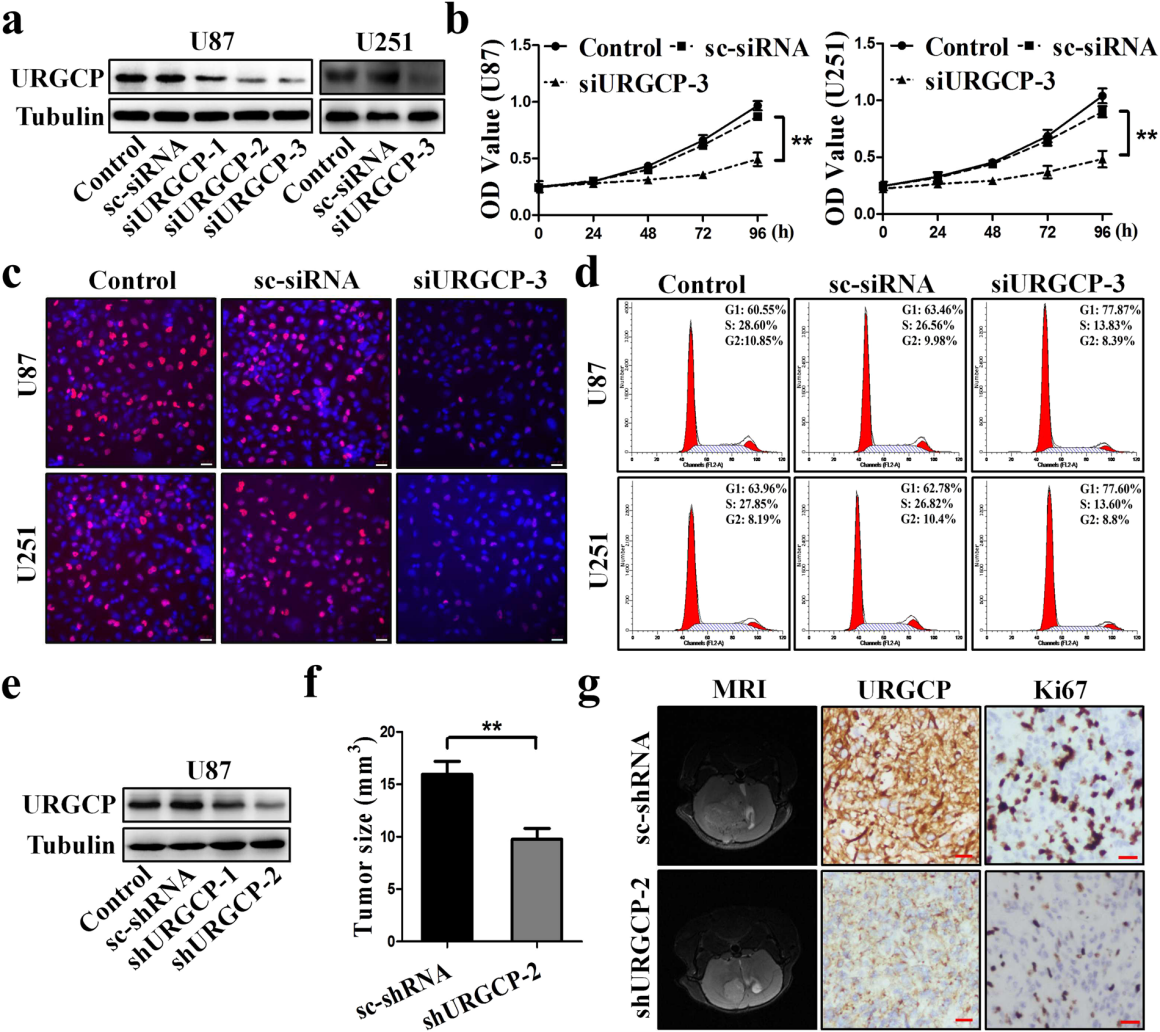
URGCP expression promotes glioma growth *in vitro* and *in vivo*. (**a**) Western blotting analysis of URGCP expression in the indicated cells. (**b**) Cell growth detected by CCK-8, the data is displayed as the means ± SD of three independent experiments, **p < 0.01. (**c**) Immunofluorescent images of EdU labeling-positive U87 and U251. Scale bars: 50 μm. (**d**) Cell cycle analysis using flow cytometry to evaluate the relative percentage of cells at G1, S and G2 phase. The data is displayed as the means ± SD of three independent experiments. (**e**) Western blotting analysis of URGCP expression in the indicated cells. (**f**) Silencing URGCP inhibited glioma growth *in vivo*. (**g**) Representative MRI images of sc-shRNA-U87 and shURGCP-2-U87 glioma growth. IHC analyses were performed for URGCP and Ki67 in glioma tissue. Scale bars: 20 μm.

To investigate the biological significance of URGCP in glioma, we first silenced URGCP in U87 cells by small interfering RNA (siRNA). We found that siURGCP-3 resulted in a better silencing effect, thus, we choose siURGCP-3 to silence URGCP in U251 cells, and for the following experiments (Fig. 2a). CCK-8 assay and EdU labeling showed that silencing URGCP suppressed the proliferative activity of U87 and U251 cells compared to control groups (Fig. 2b,c). Moreover, cell cycle analysis showed that silencing URGCP resulted in more cells in G1 phase than that in control groups (Fig. 2d). However, URGCP had no effect on the rate of apoptosis of U87 and U251 cells (Supplementary Fig. 1c).

Next, to evaluate the effects of URGCP on glioma growth *in vivo*, we first established URGCP stably silenced U87 cells by short hairpin RNA (shRNA). As shown in Fig. 2e, shURGCP-2 resulted in a better silencing effect, thus, we choose shURGCP-2-U87 cells for the *in vivo* experiments. We inoculated nude mice brains with sc-shRNA-U87 cells and shURGCP-2-U87 cells respectively, the mice injected with sc-shRNA-U87 cells had much larger tumors than those injected with shURGCP-2-U87 cells (Fig. 2f). IHC assessment of excised xenograft tissue sections showed that the numbers of proliferative cells were significantly reduced in shURGCP-2-U87 xenografts compared to sc-shRNA-U87 xenografts (Fig. 2g). In addition, mice injected with shURGCP-2-U87 cells had a much longer survival time than those injected with sc-shRNA-U87 cells (Supplementary Fig. 1d). Therefore, the above data suggest that URGCP significantly promotes glioma growth *in vitro* and *in vivo*.

### URGCP increases Cyclin D1 and Cyclin E1 expression through NF-κB pathway

**Figure 3:**
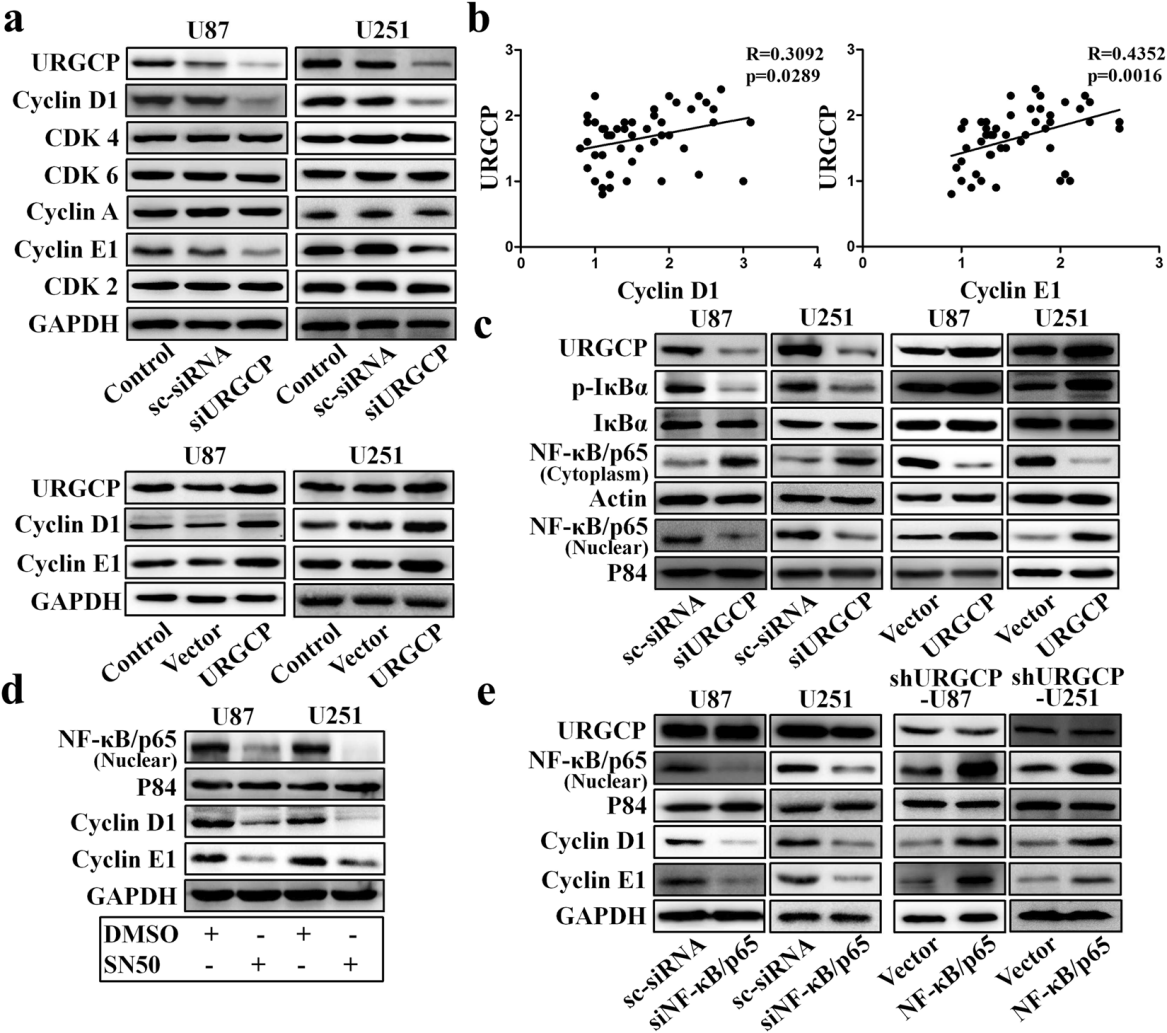
URGCP promotes Cyclin D1 and Cyclin E1 expression through NF-κB pathway. (**a**) Western blotting analysis of Cyclin D1, Cyclin E1, Cyclin A, CDK4, CDK6 and CDK2 expression in the indicated cells. (**b**) Significant positive correlations between URGCP expression (Y-axis) and Cyclin D1 and Cyclin E1 expression (X-axis) in glioma tissues. (**c**) Phospho-IκBα, total IκBα and nuclear distribution of NF-κB/p65 were analyzed by Western blotting, and P84 was used as both a marker for the nuclear fraction and a loading control. (**d**) Western blotting analysis of NF-κB/p65, Cyclin D1 and Cyclin E1 expression in the indicated cells. (**e**) Western blotting analysis of NF-κB/p65, Cyclin D1 and Cyclin E1 in the indicated cells.

Cyclin D1, Cyclin E1, Cyclin A, CDK2, CDK4 and CDK6 are the major regulators of G1/S phase transition^20^. To investigate whether URGCP regulates the expression of these regulators in glioma, we performed western blotting and found that silencing URGCP only suppressed Cyclin D1 and Cyclin E1 expression, while, URGCP overexpression enhanced Cyclin D1 and Cyclin E1 expression in U87 and U251 cells (Fig. 3a). Importantly, RT-qPCR indicated that there was less variation in the levels of Cyclin D1 and Cyclin E1 mRNA than protein under the same condition (Supplementary Fig. 2a). To test the correlations between URGCP and Cyclin D1 and Cyclin E1 expression in glioma, we first performed western blotting and found that the levels of Cyclin D1 and Cyclin E1 protein were upregulated in glioma tissues compared to normal tissues (Supplementary Fig. 2b). Indeed, we found positive correlations between URGCP and Cyclin D1 and Cyclin E1 expression in glioma tissues (Fig. 3b).

NF-κB/p65 is the major transcription factor that regulates Cyclin D1 and Cyclin E1 transcription^21,22^. To investigate whether NF-κB pathway is involved in URGCP-increased Cyclin D1 and Cyclin E1 expression in glioma, we performed western blotting. We found that silencing URGCP inhibited the phosphorylation of IκBα and the nuclear translocation of NF-κB/p65 in U87 and U251 cells, while, URGCP overexpression had opposite effects (Fig. 3c). To further confirm that NF-κB pathway is involved in URGCP-increased Cyclin D1 and Cyclin E1 expression, SN50 (NF-κB pathway inhibitor) was used to inhibit the nuclear translocation of NF-κB/p65. We found that SN50 significantly reduced the nuclear translocation of NF-κB/p65 and suppressed Cyclin D1 and Cyclin E1 expression in U87 and U251 cells (Fig. 3d). Furthermore, silencing NF-κB/p65 by siRNA also reduced Cyclin D1 and Cyclin E1 expression, while, NF-κB/p65 overexpressing abrogated the blocking effect of shURGCP on Cyclin D1 and Cyclin E1 expression in shURCGP-U87 and shURGCP-U251 cells (Fig. 3e and Supplementary Fig. 2c). Taken together, these data suggest the URGCP increases Cyclin D1 and Cyclin E1 expression at least partly by a post-transcriptional manner. Importantly, these results also suggest that NF-κB pathway is critical for URGCP-induced Cyclin D1 and Cyclin E1 expression in glioma.

### URGCP downregulates miR-16 via transcription factor c-myc

**Figure 4:**
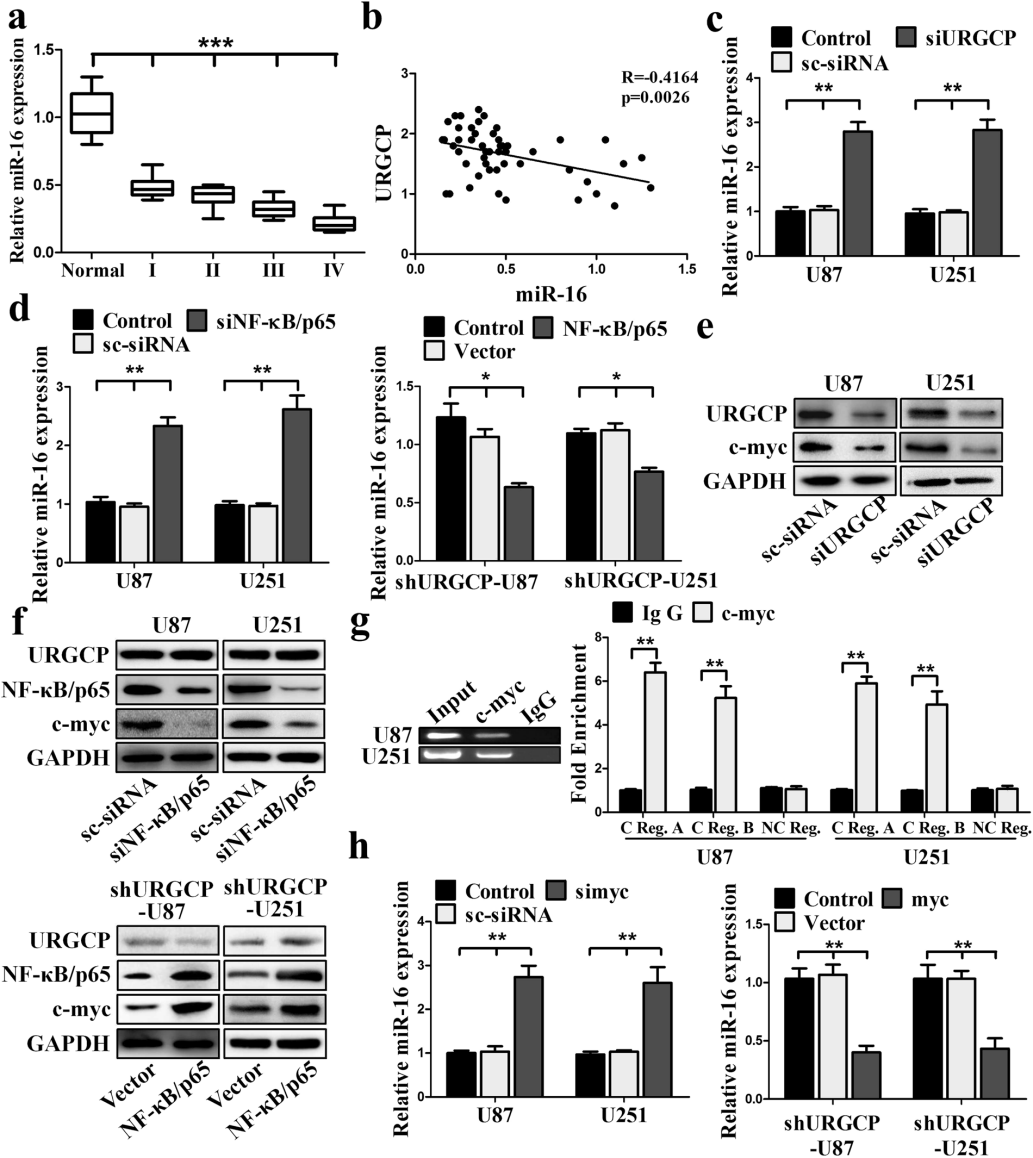
URGCP represses miR-16 expression through NF-κB/c-myc pathway. (**a**) RT-qPCR analysis of miR-16 expression in glioma tissues. (**b**) Significant negative correlation between URGCP expression (Y-axis) and miR-16 expression (X-axis) in glioma tissues. (**c**) RT-qPCR analysis of miR-16 expression in the indicated cells. (**d**) RT-qPCR analysis of miR-16 expression in the indicated cells. (**e**) URGCP and c-myc were detected by Western blotting in the indicated cells. (**f**) Western blotting analysis of NF-κB/p65 and c-myc in the indicated cells. (**g**) ChIP was performed using c-myc antibody to detect the interaction between c-myc and the promoter of miR-16. C Reg. A and C Reg. B (miR-16 promoter contains E-box), NC Reg. (approximately 6.3 kb distal from miR-16 promoter without containing E-box). (**h**) The levels of miR-16 were detected by RT-qPCR in the indicated cells. All results are expressed as means ± SD, *p < 0.05, **p < 0.01.

The above data indicates that URGCP-increased Cyclin D1 and Cyclin E1 expression is partly through a post-transcriptional manner. It has been previously established that miR-16 regulates Cyclin D1 and Cyclin E1 expression^23,24^. Therefore, we asked whether miR-16 is involved in URGCP-induced Cyclin D1 and Cyclin E1 expression in glioma. We first investigated the basic levels of miR-16 in glioma, and found that the levels of miR-16 were lower in glioma compared to normal brain tissues or astrocyte (Fig. 4a and Supplementary Fig. 3a). In addition, we also found a negative correlation between URGCP and miR-16 expression in glioma tissues (Fig. 4b). Second, to test whether URGCP regulates miR-16 expression in glioma, we silenced URGCP and found that silencing URGCP enhanced miR-16 expression in U87 and U251 cells (Fig. 4c). To investigate whether NF-κB pathway is involved in URGCP-suppressed miR-16 exprssion, we silenced NF-κB/p65 and found that silencing NF-κB/p65 elevated miR-16 expression in U87 and U251 cells; in contrast, NF-κB/p65 overexpression suppressed the expression of miR-16 in shURGCP-U251 and shURGCP-U87 cells (Fig. 4d).

Next, we investigated the underlying mechanisms of NF-κB/p65 suppresses miR-16 expression in glioma. It has been demonstrated that NF-κB/p65 is confirmed to induce the transcriptional activation of c-myc promoter in mammary cells^25^. Moreover, transcription factor c-myc represses miR-15a/miR-16 cluster expression in breast cancer and non-Hodgkin B cell lymphomas^16,23^. Therefore, we supposed that NF-κB/p65 represses miR-16 expression via c-myc. To test this, we first silenced URGCP in U87 and U251 cells, and found that knockdown URGCP repressed c-myc expression (Fig. 4e). Next, we evaluated the role of NF-κB/p65 in regulating c-myc expression, as shown in Fig. 4f, silencing NF-κB/p65 significantly decreased c-myc expression in U87 and U251 cells, while, overexpressing NF-κB/p65 markedly elevated c-myc expression in shURGCP-U87 and shURGCP-U251 cells.

Finally, to confirm c-myc regulates miR-16 expression in glioma, we first examined the direct interaction of c-myc with miR-16 promoter by chromatin immunoprecipitation assay (ChIP). We found that enforcing c-myc expression in U87 and U251 cells (Supplementary Fig. 3b) induced a 5-8-fold increase in the recruitment of c-myc to the E-box of the miR-16 promoter region rather than the region downstream of the miR-16 promoter (Fig. 4g). Next, to further confirm c-myc is critical for regulating miR-16 expression in glioma, we silenced c-myc and found that silencing c-myc elevated miR-16 expression in U87 and U251 cells (Supplementary Fig. 3c), while, overexpressing c-myc significantly suppressed miR-16 exprssion in shURGCP-U87 and shURGCP-U251 cells (Fig. 4h). These data indicate an important role of NF-κB/c-myc pathway in URGCP-repressed miR-16 expression in glioma.

### MiR-16 suppresses Cyclin D1 and Cyclin E1 expression and is involved in glioma growth

**Figure 5:**
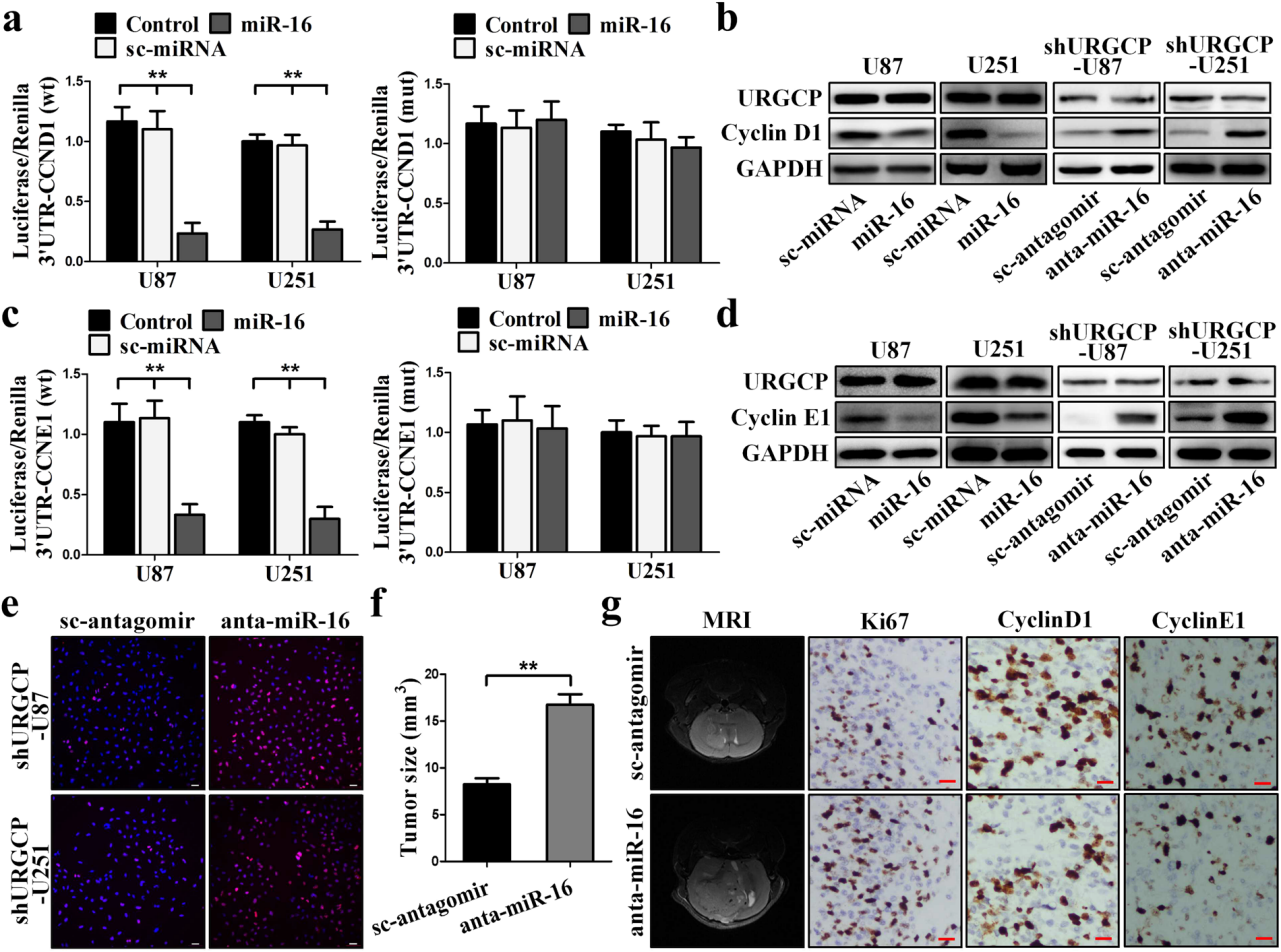
MiR-16 regulates CyclinD1 and CyclinE1 expression by targeting putative binding sites. (**a**) Luciferase assays indicated that miR-16 binds to the putative target site of CyclinD1. (**b**) Western blotting analysis of Cyclin D1 in the indicated cells. (**c**) Luciferase assays indicated that miR-16 binds to the putative target sites of CyclinE1. (**d**) Western blotting analysis of Cyclin E1 in the indicated cells. (**e**) Immunofluorescent images of EdU labeling-positive shURGCP-U87 and shURGCP-U251. Scale bars: 50 μm. (**f**) Anta-miR-16 recovered shURGCP-U87 glioma growth *in vivo*. (**g**) Representative MRI images of sc-shRNA-U87 glioma injected with sc-antagomir or anta-miR-16, IHC were performed for Ki67, Cyclin D1 and Cyclin E1. Scale bars: 20 μm. All results are expressed as means ± SD, **p < 0.01.

As shown in Supplementary Fig. 3d, the 3-untranslated region (3-UTR) of Cyclin D1 mRNA contains one highly conserved binding site for miR-16 at position 2033-2039. To investigate the relationship between miR-16 and Cyclin D1 expression in glioma, we used luciferase assays and found that overexpressing miR-16 reduced luciferase activity; however, binding sit mutation completely abolished the interaction between miR-16 and the 3′UTR of Cyclin D1 (Fig. 5a). Western blotting confirmed that overexpressing miR-16 decreased the protein levels of Cyclin D1 in U87 and U251 cells; while, transfecting anta-miR-16 restored the levels of Cyclin D1 in shURGCP-U87 and shURGCP-U251 cells (Fig. 5b). In addition, we also predicted that the 3′UTR of Cyclin E1 contains two conserved target sites for miR-16, one at position 247–253 and the other at position 484–491 (Supplementary Fig. 3d). The results of luciferase and Western blotting assays indicated that miR-16 suppressed Cyclin E1expression by interacting with these binding sites (Fig. 5c,d).

To investigate the role of miR-16 in glioma growth *in vitro*, we transfected anta-miR-16 into shURGCP-U87 and shURGCP-U251 cells, EdU labeling showed that anta-miR-16 recovered the proliferation activity of URGCP-silenced glioma cells *in vitro* (Fig. 5e). To further evaluate the effects of miR-16 on glioma growth *in vivo*, nude mice were subcutaneous injected with shURGCP-U87 and sc-shRNA-U87 cells, as shown in Supplementary Fig. 3e, mice injected with shURGCP-U87 cells had much lower tumor size than those injected with sc-shRNA-U87 cells, moreover, miR-16 also significantly suppressed the tumor growth *in vivo*. To further confirm the above data, we inoculated nude mice brains with shURGCP-U87 cells. Mice injected with anta-miR-16 had much larger tumors than those injected with sc-antagomir (Fig. 5f). In addition, IHC assessment of excised xenograft tissue sections showed that the fraction of proliferative cells and the levels of CyclinD1 and Cyclin E1 were significantly recovered in anta-miR-16 injected mice (Fig. 5g). Thus, the above data suggests that miR-16 suppresses Cyclin D1 and Cyclin E1 expression in glioma, and miR-16 plays an important role in URGCP-mediated glioma growth *in vitro* and *in vivo*.

## Discussion

URGCP is the first gene identified to be upregulated in the presence of HBxAg, which promotes the growth and survival of hepatocellular carcinoma^3^. One study has identified that URGCP is expressed at high levels in gliomas, which acts as a potential diagnostic biomarker and immunotherapeutic target for glioma patients^12^. However, the exact Biological effects of URGCP in glioma have not been precisely characterized. The data presented in this report suggests a pivotal role of URGCP in glioma growth, first, URGCP was found to be overexpressed in glioma cell lines, and in a large proportion of clinical glioma samples; second, the expression levels of URGCP correlated with the pathological grades and the overall survival time of glioma patients; third, silencing URGCP suppressed the proliferative activity of glioma *in vivo* and *in vitro*.

In this study, we detected URGCP expression in glioma specimens and showed that high-levels of URGCP positive correlated with the clinical stages of glioma, while, negative correlated with the overall survival time of glioma patients. The significance of URGCP to these clinical features suggests that URGCP may be helpful for predicting the prognosis of patients with glioma. This study also provides the first demonstration that URGCP is a positive regulator of tumor growth in glioma, while, knockdown of URGCP effectively suppresses cell proliferative activity and tumor growth via inhibiting the G1/S transition. Mechanically, overexpressing URGCP promoted Cyclin D1 and Cyclin E1 expression, however, silencing URGCP suppressed Cyclin D1 and Cyclin E1 expression, and both Cyclin D1 and Cyclin E1 are critical regulators for S phase entry in the cell cycle. Importantly, we showed that URGCP increased Cyclin D1 and Cyclin E1 expression at both the mRNA and protein levels, however, there were less variation in mRNA levels than in protein levels, suggesting the presence of post-transcriptional control. Indeed, in the follow-up experiments, we found that gliomas displayed lower levels of miR-16 compared with non-tumoral tissues and cells. We found that overexpressing URGCP suppressed miR-16 expression, while, silencing URGCP significantly upregulated miR-16 expression in U87 and U251 cells, suggesting that the low-levels of miR-16 expression is maintained at least in part by URGCP in glioma. In addition, we identified Cyclin D1 and Cyclin E1 as direct targets of miR-16 in glioma cells, overexpressing miR-16 significantly decreased Cyclin D1 and Cyclin E1 expression in glioma cells, suggesting that miR-16 is involved in URGCP-enhanced Cyclin D1 and Cyclin E1 expression in glioma. Therefore, the inhibitory effects on proliferation mediated by URGCP-knockdown are due, at least in part, to the capacity of miR-16 to inhibit Cyclin D1 and Cyclin E1 expression. And, more remarkable, URGCP also regulated the mRNA levels of Cyclin D1 and Cyclin E1 in glioma, suggesting that the transcriptional regulation is involved in this process, and the underlying mechanisms need further investigation.

It has been demonstrated that the miR-15a/miR-16-1 cluster is located on the non-coding gene deleted in leukemia 2 (DLEU2)^26^. The promoter of DLEU2 contains two c-myc binding sites (E-box)^16^. C-myc is an important oncogenic transcription factor, which is pathologically activated in many human malignancies^27^, including glioma^15^. It has been confirmed that a consequence of c-myc activation is the widespread repression or stimulation of miRNAs expression by binding to the promoters of miRNAs^28,29^. These results demonstrated that extensive reprogramming of miRNAs transcriptome by c-myc contributes to tumorigenesis. Interestingly, c-myc is reported to directly bind to the DLEU2 promoter and subsequently decrease the expression of DLEU2 and miR-16^16^. Indeed, our study also demonstrated that c-myc acts as a transcriptional repressor to inhibit miR-16 expression in glioma. The low-levels of intracellular miR-16 result in increasing Cyclin D1 and Cyclin E1 expression, and subsequently promoting glioma cells proliferation *in vitro* and *in vivo*.

Aberrant activation of NF-κB pathway is observed in a variety of tumor types, which regulates a range of tumorgenic processes, including proliferation, invasion, metastasis and angiogenesis, by transcriptionally activating numerous target genes, such as CCND1, MYC, MMP9 and VEGF in cancer cells^30,31^. Activation of NF-κB pathway is negatively regulated by the IκBs, which bind and sequester NF-κB in the cytoplasm in an inactive state. Phosphorylating IκBs, which are markers for the active NF-κB pathway, lead to their ubiquitin-mediated degradation and consequently enable the release and nuclear translocation of NF-κB/p65. Consistent with these well-studied processes, our study demonstrated that URGCP upregulates the level of p-IκBα and promotes the nuclear translocation of NF-κB/p65 in glioma cells, suggesting that the NF-κB pathway plays an essential role in the URGCP-induced glioma cell proliferation. The underlying mechanism of URGCP-induced NF-κB pathway activity in glioma cells still unclear, while, some reports indicate that URGCP increases the levels of phosphorylated IKKs in hepatocellular carcinoma cells and non-small cell lung cancer cells^8,10^. Phosphorylated IKKs leads to ubiquitin-mediated degradation of IκBs, and consequently enables the release and nuclear translocation of NF-κB/p65, suggesting that URGCP-mediated IκBα phosphorylation might through phosphorylating IKKs in glioma cells, however this hypothesis needs further investigation. In this study, we demonstrated that URGCP-mediated nuclear translocation of NF-κB/p65 induces c-myc expression, suggesting that the activation of NF-κB pathway is involved in URGCP-mediated c-myc unregulation in glioma. Our study also demonstrated that c-myc acts as a transcriptional repressor to inhibit miR-16 expression in glioma, and the low-levels of intracellular miR-16 results in glioma growth through increasing Cyclin D1 and Cyclin E1 expression.

**Figure 6:**
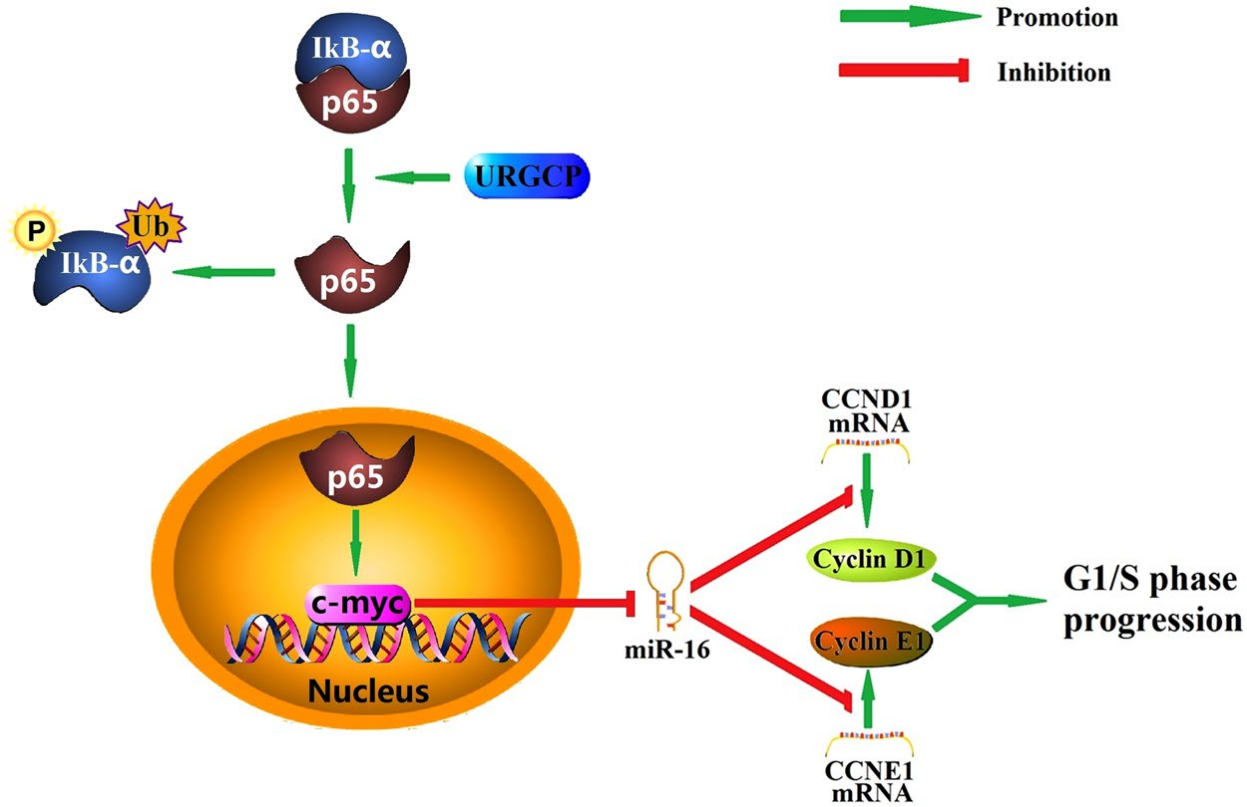
Schematic model. URGCP induces transcription factor c-myc expression by stimulating NF-κB pathway; C-myc represses miR-16 expression by binding to its promoter; CyclinD1 and CyclinE1 are the direct targets of miR-16, thus, the low-levels of miR-16 results in glioma growth through increasing Cyclin D1 and Cyclin E1 expression in glioma.

In conclusion, the results of this study demonstrate that URGCP increases transcription factor c-myc expression through activating the classical NF-κB pathway in glioma; Transcription factor c-myc suppresses miR-16 expression through binding to the promoter of miR-16; Cyclin D1 and Cyclin E1 are indentified as the direct targets of miR-16, thus, low-levels of miR-16 leads to G1/S phase transition and tumor growth by enhancing the expression of Cyclin D1 and Cyclin E1 *in vivo* and *in vitro* (Fig. 6). In addition to identifying a new mechanism of action for URGCP in glioma, our results also suggest that URGCP and miR-16 may be considered as candidates for targeted glioma treatment. A miR-16-based treatment may have the potential to target multiple genes, such as CCND1 and CCNE1, thereby amplifying the antiproliferative response.

## Methods

### Cell culture and miRNAs transfection

Glioma cell lines U87 and A172 were supplied by the American Type Culture Collection and maintained in DMEM (Invitrogen, Carlsbad, CA) and supplemented with 10% FBS (Hyclone, Logan, UT) and 1% penicillin/streptomycin (Invitrogen). Primary glioma cell and primary astrocyte were isolated from GBM sample and non-tumoral brain tissue. miR-16 mimic, anta-miR-16, sc-miRNA and sc-antagomir were purchased from RIBOBIO (CHN) and transfected in accordance with the manufacturer’s instruction. Details are described in supplementary methods.

### Antibodies and western blotting

The following antibodies were used for Western blotting: anti-URGCP, anti-β-actin, anti-β-tubulin and anti-GAPDH (Sigma), anti-CDK2, anti-CDK4, anti-CDK6, anti-Cyclin D1, anti-Cyclin E1, anti-c-myc, anti-p65, anti-phosphor-IκBα and anti-P84 (Cell signaling, Danvers, MA), anti-Cyclin A (Santa Cruz, CA, USA). Details are described in supplementary methods.

### Flow cytometry

Cultured cells were subjected to the different treatments. For cell cycle assay, cells were harvested and washed twice with cold PBS, followed by fixation with cold 70% ethanol overnight at 4 °C. After washing twice with PBS, cells were incubated with PI and RNaseA. Cells were analyzed using a FACS C6 with Cell Quest analysis software. For apoptosis assay, cells were collected and washed twice with cold PBS, resuspended in 0.1 ml of binding buffer, incubated with 5 μl Annexin V conjugated to FITC and 10 μl PI, then analyzed with Cell Quest analysis software.

### siRNA and shRNA transfections

siRNAs targeting URGCP, NF-κB/p65 and c-myc were purchased from RIBOBIO. A nonsilencing siRNA oligonucleotide that didn’t target any mammalian gene was used as a negative control. Transfection of siRNA duplexes was performed using the transfection reagent (Invitrogen) according to the manufacturer’s instruction. shURGCP Lentiviral particle was purchased from GENECHEM (CHN), and a control shRNA was used as control. U87 and U251 were transfected with shURGCP and selected with puromycin (Sigma).

### Tissue specimens and Immunohistochemistry (IHC)

Clinical glioma samples (WHO I-IV) were histopathologically and clinically diagnosed at the Daping hospital of Third Military Medical University. Non-tumoral brain specimens were taken from a standard distance from the margin of resected neoplastic tissues of patients with glioma who underwent surgery. IHC procedure to detect URGCP (Antibody: Sigma (human) and Shangon biotech (mice)) and Ki67 were performed using CECTASTAIN ABC Detection system (VECTOR) according to the manufacturer’s instruction. The results of URGCP and Ki67 detection were determined according to the percentage of positive cell number to total number in 10 randomly selected microscopic fields.

### Real time quantitative RT-PCR (RT-qPCR)

Level of miR-16 was analyzed by RT-qPCR using a TaqMan®MicroRNA assay specific for miR-16 of human according to the manufacturer’s instruction. Primers were purchased for RIBOBIO, U6 was used as the normalization procedure. To test the levels of Cyclin D1 and Cyclin E1 mRNA, total RNA was isolated using TRIzol reagent (Invitrogen). cDNA was amplified by RT-qPCR performed with an ABI Prism 7500 sequence detector using SYBR green PCR master mix (Applied Biosystems, CA, USA). GAPDH was chosen as the normalization procedure. Details are described in supplementary methods.

### Xenografted glioma model, MRI and HE staining

Ten male NOD/SCID mice (4 weeks of age, 12–15 g) were divided into two groups: control group (n = 5), sc-shRNA-U87 cells were injected into mice brains; shURGCP group (n = 5), shURGCP-U87 cells were injected into mice brains (2.5 mm lateral, 0.4 mm frontal of bregma and 3.5 mm deep from skull). Mice were imaged MRI at 9 days after injection, and every 4 day until 21 days after injection. Tumor volumes were calculated as Length × Width^2^ × 0.52. HE staining was performed on sections of paraffin embedded mice brains for histological confirmation of glioma. All surgery was performed under anesthesia with sodium pentobarbital.

### Plasmid and Luciferase plasmid construction

Human RALA/p65, URGCP and c-myc cDNAs were purchased from OriGene (USA) and inserted into pLenti-C-mGFP vectors. The 3′UTR of CCND1 mRNA contains one binding site for miR-16 (2033–2039 bp), the 3′UTR of CCNE1 mRNA contains two binding site (247–253 and 484–491 bp). The luciferase constructs carrying the wide type or mutated response sites for CCND1 and CCNE1 were purchase from RIBOBIO (pmiR-RB-ReportTM Vector).

### Chromatin immunoprecipitation (ChIP)

DNA-protein complexes were immunoprecipitated from U87 and U251 after c-myc overexpression vector transfection using the ChIP Kit (Millipore, MA, USA) according to the manufacturer’s protocol with 1 mg polyclonal antibody c-myc or normal IgG (Millipore). Details are described in supplementary methods.

### Ethics approval and consent to participate

For the use of clinical materials for research purposes, written informed consents were obtained from all patients and healthy controls. All methods were performed in accordance with the guidelines and regulations of the Ethics Committees of Third Military Medical University. Animal experiments were carried out in strict accordance with the guide for the care and use of laboratory animals published by the National Institutes of Health (NIH). The whole protocol was approved by the Ethics Committee of Daping Hospital, Third Military Medical University.

### Statistical Analysis

All statistical analyses were carried out using SPSS 13.0 statistical software packages. Comparisons between two groups were performed using Student’s t test. Survival curves were plotted by the Kaplan-Meier method using the log-rank test. P value less than 0.05 was considered statistically significant in all cases.

## Electronic supplementary material

supplementary material
