# Supplementary material for: Downregulation of miR-16 via URGCP pathway contributes to glioma growth
Source: Sci Rep. 2017 Oct 18;7:13470. doi: 10.1038/s41598-017-14035-2 (PMC5647380; doi:10.1038/s41598-017-14035-2)
Supplement: Supplementary file 1 — supplementary material [file 41598_2017_14035_MOESM1_ESM.pdf]

Downregulation of miR-16 via URGCP pathway contributes to glioma growth

Liang Hong[1,#]; Ouyang Qing[1,#]; Zhou Ji[2]; Zhang Chengqu[1]; Chen Ying[1]; Cui Hao[3]; Xu Minhui[1,*]; Xu Lunshan[1,*]

Supplementary Materials and Methods

Cell culture and miRNAs transfection

Sample was dissected into 1 mm fragments and mechanically dissociated into a single cell suspension through consecutive 100 μm, 70 μm and 40 μm cell strainers. Cells were cultured in RPMI-1640 with 10% FBS, 1% penicillin/streptomycin, and 1% glutamax (Sigma, Saint Louis, MI). Media was changed 2 days postplating to remove debris.

Antibodies and western blotting

The nuclear proteins were extracted using CelLytic NuCLEAR Extraction Kit (Sigma) according to the manufacturer's instruction. Lysates were prepared from cells subjected to the different treatment and proteins were resolved by SDS-PAGE as described. Membranes were immunoblotted with the antibodies described in each experiment and filters were reprobed, after stripping, with antibodies against P84, β-actin, β-tubulin and GAPDH as loading control.

Real time quantitative RT-PCR (RT-qPCR)

The primers:

CCND1-F, 5'-CTGTGCATCTACACCGACAACT-3'

CCND1-R, 5'-GCATTTTGGAGAGGAAGTGTTC-3'

CCNE1-F, 5'-CACCACTGAGTGCTCCAGAA-3'

CCNE1-R, 5'-CTGTTGGCTGACAGTGGAGA-3'

GAPDH-F, 5'-AGGTCCACCACTGACACGTT-3'

GAPDH-R, 5'-GCCTCAAGATCATCAGCAAT-3'

Chromatin immunoprecipitation (ChIP)

Cells were lysed, disrupted by sonication, and cleared by centrifugation. Precipitated

DNA was subjected to qPCR analysis by using specific primers, data were analyzed using the 2 (-Delta Delta C(C)) method. Contain Region (C Reg.) primers were used to amplify the genomic region encompassing the E-box (c-myc binding sequence). Non-Contain Region (NC Reg.) primers were used to amplify the region, 6.3 kb distal from the transcription start of promoter. The primers:

Contain Region-F, 5'-ACGGCAAAAGCTCTACAAGC-3'

Contain Region-R, 5'-GGGTCCTGCTTAGGAGAAAA-3'

Non-Contain Region-F, 5'-ACTGTGGAACTGCAGGAGATGGA-3'

Non-Contain Region-R, 5'-CTCTGCTACAGGTCAAAACCTGCA-3'

Supplementary Figures and Legends

Figure S1

(a) Box plot of URGCP levels in different pathological types of glioma and different grades in the Sun, Harris and Hegi dataset, *$p<0.05$. (b) Kaplan-Meier analysis of overall survival according to URGCP expression for the Gravedeel dataset, p valve was determined using the log-rank test. (c) Representative flow cytometry profiles and percentages of apoptotic cells. (d) Survival curves for mice injected with sc-shRNA-U87 and shURGCP-2-U87 cells over the 40-day experiment period.

Figure S2

(a) RT-qPCR analysis of Cyclin D1, CDK4, CDK6 Cyclin E1, Cyclin A and CDK2 mRNA expression in the indicated cells, *$p<0.05$. (b) The levels of Cyclin D1 and Cyclin E1 expression in glioma tissues. (c) RT-qPCR were performed to detect the relative mRNA expression of Cyclin D1 and Cyclin E1 in the indicated cells, *$p<0.05$.

Figure S3

(a) Expression of miR-16 in primary astrocyte, primary glioma cell and glioma cell lines (U251 and U87). Bars represent the means ± SD of three independent experiments, **$p<0.01$. (b) Western blotting analysis of c-myc expression in the indicated cells. (c) Western blotting analysis of c-myc expression in the indicated cells. (d) Sites of complementarity sequences between miR-16 and Cyclin D1 and Cyclin

E1 mRNA. (e) Silencing URGCP or transfecting miR-16 inhibited glioma growth in *vivo*, Representative images of U251 glioma growth. Scale bars: 1 mm.

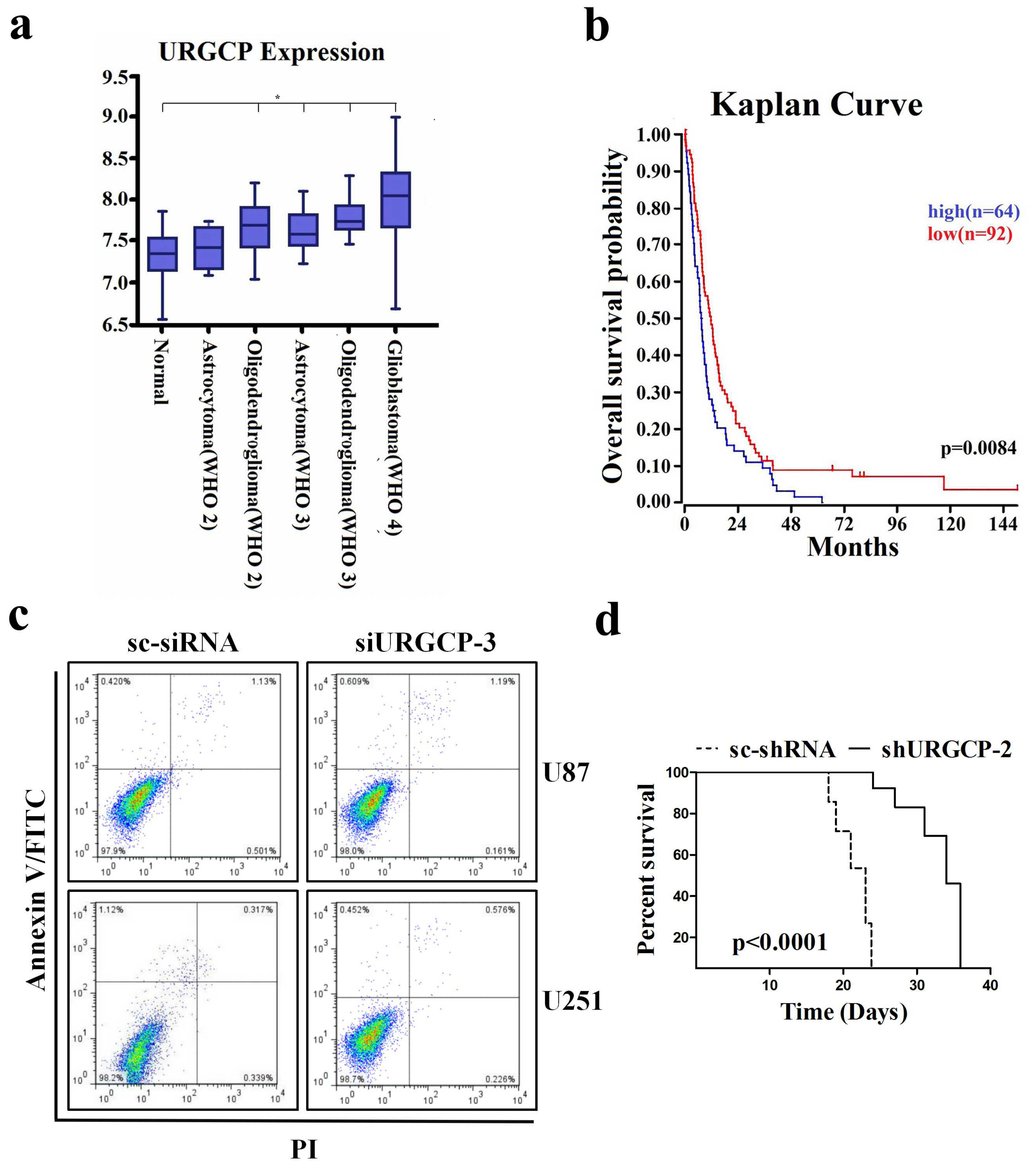
a
URGCP Expression
9.5
9.0
8.5
8.0
7.5
7.0
6.5
*
Normal
Astrocytoma(WHO 2)
Oligodendroglioma(WHO 2)
Astrocytoma(WHO 3)
Oligodendroglioma(WHO 3)
Glioblastoma(WHO 4)
b
Kaplan Curve
Overall survival probability
1.00
0.90
0.80
0.70
0.60
0.50
0.40
0.30
0.20
0.10
0.00
high(n=64)
low(n=92)
p=0.0084
0
24
48
72
96
120
144
Months
c
sc-siRNA
siURGCP-3
U87
U251
Annexin V/FITC
PI
0.420%
1.13%
97.9%
0.501%
0.609%
1.19%
98.0%
0.161%
1.12%
0.317%
98.2%
0.339%
0.452%
0.576%
98.7%
0.226%
d
sc-shRNA
shURGCP-2
Percent survival
100
80
60
40
20
p<0.0001
10
20
30
40
Time (Days)

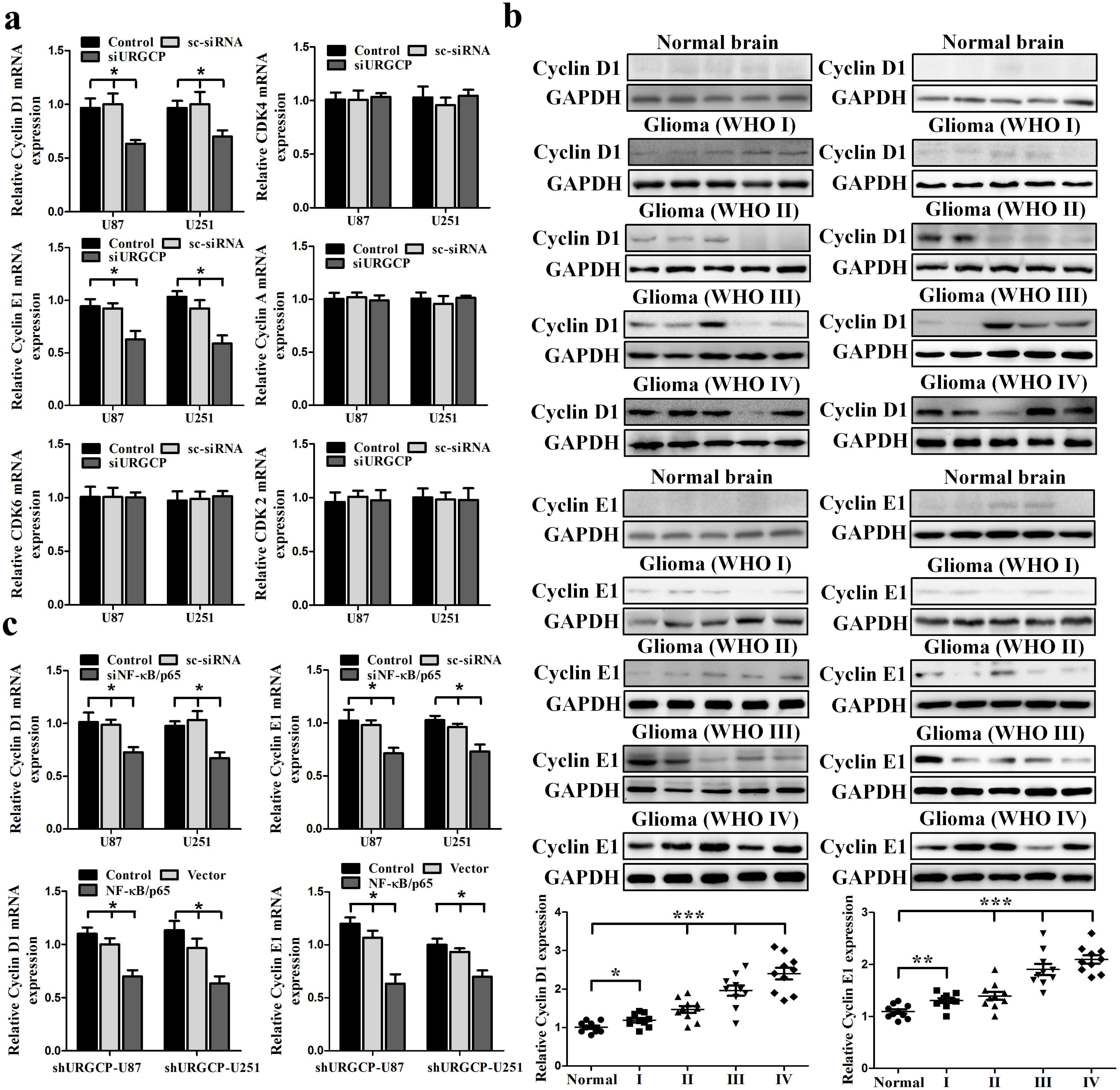
a
Control
sc-siRNA
siURGCP
Relative Cyclin D1 mRNA expression
Relative CDK4 mRNA expression
Relative Cyclin E1 mRNA expression
Relative Cyclin A mRNA expression
Relative CDK6 mRNA expression
Relative CDK 2 mRNA expression
U87
U251
b
Normal brain
Glioma (WHO I)
Glioma (WHO II)
Glioma (WHO III)
Glioma (WHO IV)
Cyclin D1
Cyclin E1
GAPDH
Relative Cyclin D1 expression
Relative Cyclin E1 expression
Normal
I
II
III
IV
c
siNF-κB/p65
Vector
NF-κB/p65
shURGCP-U87
shURGCP-U251

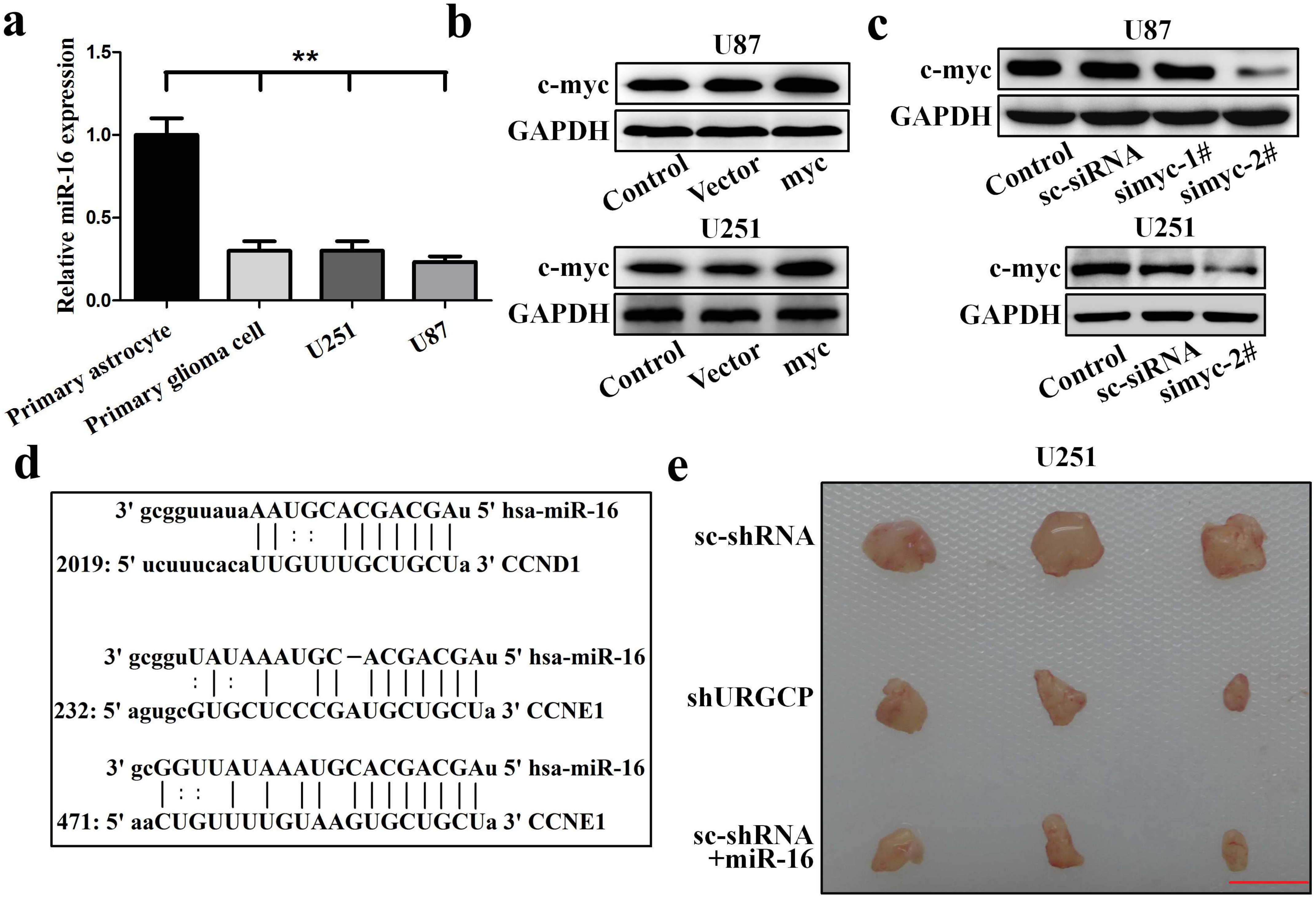
a
Relative miR-16 expression
1.5
1.0
0.5
0.0
**
Primary astrocyte
Primary glioma cell
U251
U87
b
U87
c-myc
GAPDH
Control
Vector
myc
U251
c-myc
GAPDH
Control
Vector
myc
c
U87
c-myc
GAPDH
Control
sc-siRNA
simyc-1#
simyc-2#
U251
c-myc
GAPDH
Control
sc-siRNA
simyc-2#
d
3' gcgguuauaAAUGCACGACGAu 5' hsa-miR-16
2019: 5' ucuuucacaUUGUUUGCUGCUa 3' CCND1
3' gcgguUAUAAAUGC−ACGACGAu 5' hsa-miR-16
232: 5' agugcGUGCUCCCGAUGCUGCUa 3' CCNE1
3' gcGGUUAUAAAUGCACGACGAu 5' hsa-miR-16
471: 5' aaCUGUUUUGUAAGUGCUGCUa 3' CCNE1
e
U251
sc-shRNA
shURGCP
sc-shRNA
+miR-16
